# The impact of the RBM4-initiated splicing cascade on modulating the carcinogenic signature of colorectal cancer cells

**DOI:** 10.1038/srep44204

**Published:** 2017-03-09

**Authors:** Jung-Chun Lin, Yuan-Chii Lee, Yu-Chih Liang, Yang C. Fann, Kory R. Johnson, Ying-Ju Lin

**Affiliations:** 1 School of Medical Laboratory Science and Biotechnology, College of Medical Science and Technology, Taipei Medical University, Taipei, Taiwan; 2 Graduate Institute of Biomedical Informatics, Taipei Medical University, Taipei, Taiwan; 3 Information Technology and Bioinformatics Program, Division of Intramural Research, National Institute of Neurological Disorders and Stroke, National Institutes of Health, Bethesda, MD, USA; 4 School of Chinese Medicine, China Medical University, Taichung, Taiwan

## Abstract

A growing body of studies has demonstrated that dysregulated splicing profiles constitute pivotal mechanisms for carcinogenesis. In this study, we identified discriminative splicing profiles of colorectal cancer (CRC) cells compared to adjacent normal tissues using deep RNA-sequencing (RNA-seq). The RNA-seq results and cohort studies indicated a relatively high ratio of exon 4-excluded *neuro-oncological ventral antigen 1 (Nova1*^−*4*^) and intron 2-retained *SRSF6 (SRSF6*^*+intron 2*^) transcripts in CRC tissues and cell lines. Nova1 variants exhibited differential effects on eliminating SRSF6 expression in CRC cells by inducing *SRSF6*^*+intron 2*^ transcripts which were considered to be the putative target of alternative splicing-coupled nonsense-mediated decay mechanism. Moreover, the splicing profile of *vascular endothelial growth factor (VEGF)165/VEGF165b* transcripts was relevant to SRSF6 expression, which manipulates the progression of CRC calls. These results highlight the novel and hierarchical role of an alternative splicing cascade that is involved in the development of CRC.

Alternative splicing constitutes a major mechanism in expanding the proteome diversity of eukaryotic cells^1^. Recent studies demonstrated that more than 90% of protein-coding genes generate more than one transcript through this meticulously controlled process^2^. In normal tissues, spatiotemporal expression profiles of alternative splicing events determine cell differentiation and specification^3^, whereas imbalanced or aberrant alternative splicing profiles are highly relevant to hereditary diseases and cancers^4^,^5^. The interplay between splicing factors, such as serine-arginine (SR) or the heteronuclear ribonucleoprotein (hnRNP) protein family, and *cis*-elements within cassette exons of alternative splicing events, constitutes the molecular mechanism of programming splicing profiles^6^. Altered expressions or distributions of splicing factors were subsequently noted to be an efficient means for reprogramming splicing profiles of cancer cells^7^,^8^.

Statistical studies indicated that while colorectal cancer (CRC) is the third most common and lethal cancer in the human race, the etiology and molecular mechanisms leading to CRC are largely unclear^9^. However, several alternative splicing events are widely observed and relevant to the development of CRC^10^. For instance, upregulation of SRSF10 expression results in an increase in BCLAF1 exon 5a-included transcripts which facilitates the progression of CRC^11^. In addition, an increase in the expression of polypyrimidine tract-binding protein 1 (PTBP1) leads to a relatively high level of the PKM2 variant which mediates the Warburg effect in CRC cells^12^. Our previous work reported that overexpression of RNA-binding motif protein 4 (RBM4) constitutes a regulatory network in reprogramming splicing profiles of the *FGFR2* and *PKM* genes, which modulate the progression and metabolic signature of CRC cells^13^.

With the development of high-throughput approaches, including deep RNA sequencing (RNA-seq) and RNA crosslink immunoprecipitation-coupled proteomic analyses, alternative splicing networks can be investigated on a genome-wide level^14^,^15^. In this study, we performed RNA-seq analyses to thoroughly annotate transcriptomes in CRC tissues and adjacent normal tissues. Among these alternative splicing events, RBM4, Nova1, SRSF6, and vascular endothelial growth factor 165 (VEGF165) comprised a splicing cascade in a consensus sequence-dependent manner which manipulated the migration and angiogenetic signature of CRC cells. These results suggested the potential value of splicing events as clinical applications for CRC treatment.

## Results

### Genome-wide analyses of CRC-associated alternative splicing events

**Figure 1:**
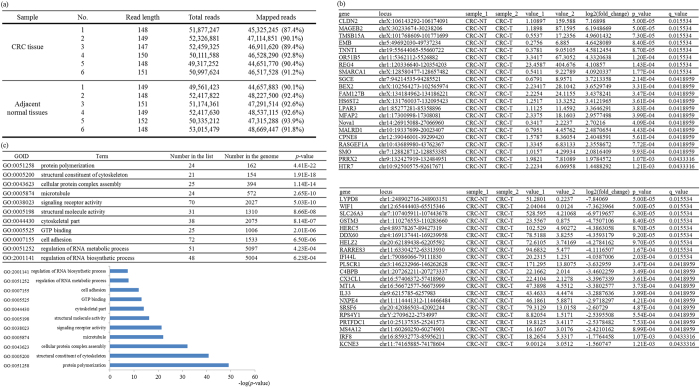
Transcriptome and gene ontology analyses of paired colorectal cancer (CRC) tissues. (**a**) Statistical summaries of transcriptome analytical results. (**b**) Transcriptome analysis-identified splicing events significantly changed in CRC tissues compared to adjacent normal tissues. (**c**) The top 11 enriched functions for differentially spliced genes in CRC tissues.

Disclosing an imbalanced or aberrant splicing event may bring about a comprehensive understanding of the genetic causes of distinct malignancies. We therefore performed transcriptome analyses using deep RNA-seq with total RNAs extracted from cancerous and adjacent normal tissues of anonymous CRC patients. The statistical summary of the RNA-seq output showed close numbers of read lengths, amplified reads, and mapping rates within the independent analyses (Fig. 1a, *n *=* *6). After filtering differentially spliced transcripts with a false detection rate (FDR)-adjusted *p* value of <0.01, a *q* value of <0.05, and a multiple of change of >2, 153 genes from a total of 22,518 splicing events that significantly differed in cancerous tissues compared to adjacent normal counterparts were identified. Figure 1b presents 42 genes which exhibited differential splicing profiles between CRC cancerous tissues and adjacent normal tissues. For instance, the relative level of the *Nova1*^−*4*^ transcript was significantly upregulated in cancerous tissues (Fig. 1b, 2.2237) compared to adjacent normal tissues (Fig. 1b, 0.3417). In contrast, relative expression of the non-coding *SRSF6* transcripts in cancerous tissues (Fig. 1b, 79.3127) was more abundant than that of adjacent normal tissues (Fig. 1b, 13.0158). Gene ontology (GO) analyses were next conducted by examining putative CRC-related splicing events. Figure 1c shows the analytical summary (upper) and the bar chart (lower) presents the *p* values of the GO analyses. Results showed the enrichment of CRC-related splicing events involved in cytoskeletal, signaling receptor, and cell adhesion terms, which are highly related to the progression of cancer cells. Moreover, enriched terms corresponding to RNA metabolism and biosynthesis also implied a potential impact of RNA-binding proteins, including Nova1 and SRSF6 genes (Fig. 1b), on the carcinogenic signature of CRC cells.

### Differential expressions and splicing profiles of Nova1 and SRSF6 in CRC tissues

**Figure 2:**
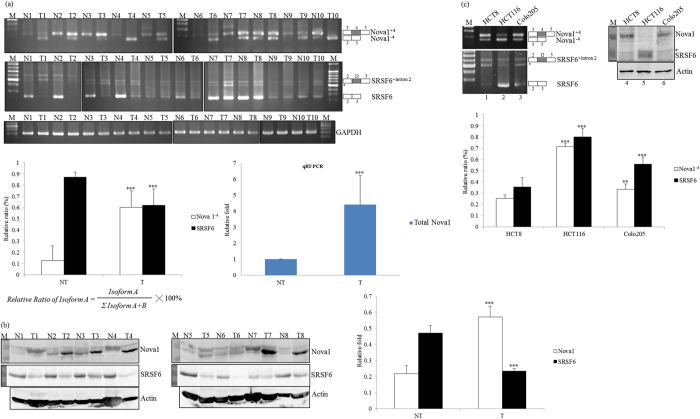
Differential splicing and expression profiles of Nova1 and SRSF6 in colorectal cancer (CRC) tissues and derived cell lines. (**a**) Total RNAs prepared from human CRC tissues (T) and adjacent non-tumorous tissues (NT) were subjected to RT-PCR and RT-qPCR analyses with specific primer sets complementary to the *Nova1* and *SRSF6* transcripts. The bar graphs present relative levels of the *Nova1* and *SRSF6* transcripts (left) or total *Nova1* transcripts (right) in paired CRC tissues (*n* = 10). (**b**) Total lysates prepared from human CRC tissues (T) and adjacent non-tumorous tissues (NT) were analyzed using an immunoblot assay with the indicated antibodies. The bar graph presents relative levels of Nova1 and SRSF6 proteins in paired CRC tissues (*n* = 8). (**c**) Total RNAs and lysates prepared from CRC-derived cell lines were analyzed by RT-PCR assays with a specific primer set against the *Nova1* and *SRSF6* genes. Western blotting was performed with the indicated antibodies. The bar graph presents relative levels of the *Nova1*^*+4*^ and *SRSF6* transcripts in three independent experiments. The gels shown in this figure were run under the same conditions and were not artificially manipulated. The signal densities were analyzed using TotalLab Quant Software, and the quantitative results are shown as the mean ± STD (*p < 0.05; **p < 0.01; ***p < 0.005).

To validate the identified results regarding the splicing profiles of *Nova1* and *SRSF6* genes, RT-PCR analyses were conducted with RNAs prepared from cancerous tissues and adjacent normal tissues of anonymous CRC patients (Fig. 2a, *n* = 10). Figure 2a shows the predominant expression of exon 4-excluded *Nova1* transcripts (*Nova1*^−*4*^, 60.1%, white bar) in cancerous tissues, whereas the reduced expression of *Nova1*^−*4*^ transcript (12.5%, white bar) was noted in the adjacent normal tissues of CRC patients. Furthermore, results of the RT-qPCR showed elevated total *Nova1* transcripts in cancerous tissues compared to adjacent normal tissues (by about 4.4-fold; Fig. 2a, qRT-PCR). In addition to *Nova1*, RNA-seq results also indicated differential splicing profiles of the *SRSF6* gene between normal and cancerous tissues (Fig. 1b, lower table). The RT-PCR results showed that adjacent normal tissues exhibited predominant expression of authentic *SRSF6* transcripts (Fig. 2a, NM_006275; 87%, black bar) encoding functional SRSF6 proteins, whereas splicing profiles of the *SRSF6* gene in cancerous tissues were shifted to non-coding *SRSF6* transcripts (NR_034009, 38%, black bar), which are the potential target of the nonsense-mediated decay (NMD) mechanism^16^. As expected, the analytical results of immunoblotting revealed upregulated expression of the Nova1 protein (by about 2.64-fold) in cancerous tissues (Fig. 2b, white bar). The abundance of the SRSF6 protein was closely related to the relative level of coding *SRSF6* transcripts in paired CRC tissues (Fig. 2b, black bar). In addition, differential splicing profiles of *Nova1* and *SRSF6* were noted in distinct CRC cell lines. The RT-PCR results indicated close expressions of *Nova1*^−*4*^ (Fig. 2c, 25.2% and 33.5%, white bar) and *SRSF6* transcripts (Fig. 2c, 35.5% and 56.1%, black bar) in the HCT8 and Colo205 colorectal adenocarcinoma cell lines, respectively. In contrast, predominant expressions of the *Nova1*^−*4*^ (Fig. 2c, 71.5%, white bar) and authentic *SRSF6* transcripts (Fig. 2c, 80.2%, black bar) were observed in HCT116 cells derived from colorectal carcinoma. Expression profiles of the *Nova1* and *SRSF6* transcripts were highly relevant to the encoded proteins in distinct CRC cells (Fig. 2c, right panel). The molecular mechanisms contributing to the differential splicing profiles of *Nova1* or *SRSF6* in different CRC cell lines are worthy of further investigation.

### RBM4 and Nova1 constitute an antagonistic circuit toward the selection of Nova1 exon 4

**Figure 3:**
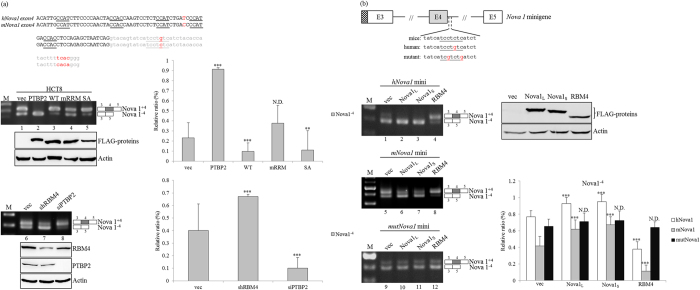
RBM4 and Nova1 exert differential effects on splicing profiles of the *Nova1* gene. (**a**) The diagram presents the sequence of human and mouse *Nova1* exon 4 and flanking introns. The responsive elements of Nova1 (underlined black characters) and RBM4 (underlined gray characters) are underlined. HCT-8 cells were transfected with overexpressing or targeting vectors, followed by total RNA and lysate extraction. Splicing profiles of *Nova1* transcripts were analyzed with specific primer sets. Immunoblot assays of indicated proteins were analyzed with specific antibodies. The bar graph shows relative levels of *Nova1*^−*4*^ in three independent experiments. (**b**) *Nova1* minigenes were cotransfected with expressing vectors into HCT-8 cells. Spliced transcripts of *Nova1* minigenes were analyzed using SV40 oligo and a specific *Nova1* primer as described in the previous panel. The bar graph shows the relative level of *Nova1*^−*4*^ in three independent experiments using TotalLab Quant Software. The quantitative results are shown as the mean ± STD (*p* < 0.05; **p < 0.01; ***p < 0.005). The gels shown in this figure were run under the same conditions and were not artificially manipulated.

Post-transcriptional mechanisms, including alternative splicing and mRNA turnover, constitute multilayer control for regulating gene expressions^17^. In our previous study, overexpression of the PTBP2 protein exhibited multiple functions related to the stability and splicing profile of the *Nova1* gene, which abolishes brown adipogenesis^18^. The relatively high expression of PTBP2 with a concomitant reduction in the RBM4 protein was documented in HCT8 and Colo205 cells compared to that of HCT116 cells^13^, which may also manipulate splicing profiles of the *Nova1* and *SRSF6* genes (Fig. 2c). The alignment results showed high identity between human and mouse *Nova1* exon 4 and the downstream intron containing multiple YCAY motifs (Fig. 3a), although the intronic CU element next to human *Nova1* exon 4 was shortened by interruption with a single guanine residue (Fig. 3a, gray characters). The RT-PCR results revealed the effect of overexpressing PTBP2 on enhancing relative levels of *Nova1*^−*4*^ transcripts (Fig. 3a, lane 2), whereas relatively high expression levels of *Nova1*^+*4*^ transcripts were observed with overexpression of RBM4 and the derived mutant containing the serine-to-alanine (SA) substitution in HCT8 cells (Fig. 3a, lanes 3 and 5). Nevertheless, the splicing profile of *Nova1* showed no response to the presence of an RNA recognition motif (RRM) mutant (Fig. 3a, lane 4), consistently implying the influence of RBM4 on programming splicing profiles through a post-transcriptional mechanism^16^. Inversely, depletion of endogenous RBM4 upregulated relatively high levels of *Nova1*^−*4*^ transcripts (Fig. 3a, lane 7), whereas a reduction in PTBP2 mediated an increase in *Nova1*^*+4*^ transcripts (Fig. 3a, lane 8).

The interplay between RBM4 and the intronic CU element next to *Nova1* exon 4 enhances its inclusion in murine brown adipocytes^16^. Moreover, Nova1 isoforms constitute an autoregulatory mechanism on its splicing profiles^19^. To evaluate the effects of RBM4 and Nova1 variants on the human *Nova1* gene, the *human Nova1* minigene containing the cytosine-to-guanine nucleotide substitution within the intronic CU element was derived from the mouse *Nova1* minigene (Fig. 3b, human). The derived mutant containing two cytosine-to-guanine nucleotide substitutions within the intronic CU element was applied in our previous study^16^. *In vivo* splicing results showed that more *Nova1*^*+4*^ transcripts were generated from the mouse *Nova1* minigene compared to that of the human *Nova1* reporter (Fig. 3b, lanes 1 and 5). Nevertheless, overexpression of RBM4 enhanced relatively high levels of *Nova1*^*+4*^ transcripts produced from both human and mouse *Nova1* minigenes (Fig. 3b, lanes 4 and 8). Overexpressing Nova1_S_ variants exerted a more-prominent effect (Fig. 3b, lanes 3 and 7; 95.2% and 67.2%) than that of Nova1_L_ variants (Fig. 3b, lanes 2 and 6; 92.9% and 61.8%) on enhancing the relative level of *Nova1*^−*4*^ transcripts generated from mouse or human *Nova1* minigenes. The mutant minigene containing two cytosine-to-guanine nucleotide substitutions within the intronic CU element showed an insubstantial response to the overexpression of RBM4 or Nova1 variants (Fig. 3b, lanes 10 ~ 12). Collectively, RBM4 constitutes a regulatory mechanism on the utilization of *Nova1* exon 4 through the intronic CU element.

### RBM4 and Nova1 exert differential effects on the migration of CRC cell lines

**Figure 4:**
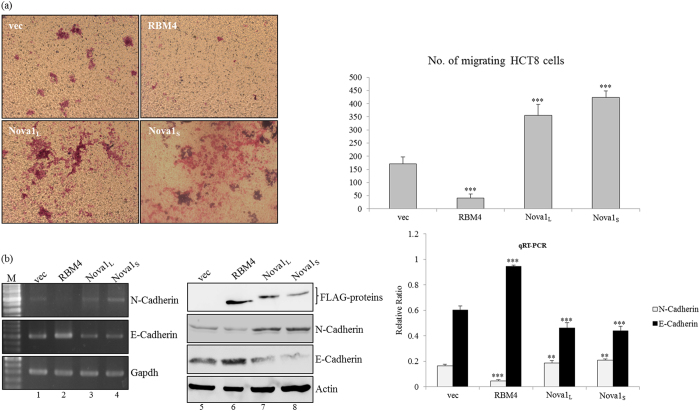
Overexpressing RBM4 and Nova1 variants differentially modulate the migratory activity of colorectal cancer (CRC) cells. (**a**) The motility of HCT-8 cells transfected with RBM4 or Nova1 variant-expressing vectors was analyzed using an *in vitro* migration assay with a transwell system, followed by Giemsa staining. The counting results of three independent experiments are presented in a bar chart as the mean ± STD. (**b**) Total RNAs and lysates were prepared from HCT-8 cells transfected with RBM4- or Nova1-expressing vectors. Expression profiles of cadherin genes and the loading control were analyzed by RT-PCR, RT-qPCR, and immunoblot assays with specific primer sets and antibodies against indicated targets. Western blotting was performed with the indicated antibodies. The gels shown in this figure were run under the same conditions and were not artificially manipulated. The bar graph presents relative levels of each target in three independent RT-qPCR analyses. Signal densities were analyzed using TotalLab Quant Software and the quantitative results are shown as the mean ± STD (*p < 0.05; **p < 0.01; ***p < 0.005).

RBM4 was documented to reduce immortalization, proliferation, and migration of distinct cancer cells by programming the related splicing profiles^20^. In contrast, upregulation of Nova1 expression is closely associated with the progression of astrocytomas, hepatocellular carcinoma, and gastric cancer^21^,^22^. *In vitro* migration assays were conducted to demonstrate the phenomenon in CRC cell lines. Compared to the empty vector-transfected counterparts, RBM4-overexpressing cells exhibited less migratory activity (Fig. 4a, RBM4). Contrarily, overexpression of Nova1 variants enhanced the migratory activity of HCT8 cells (Fig. 4a, Nova1_S_ and Nova1_L_). Furthermore, imaging and statistical results indicated a more-potent effect of the Nova1_S_ isoform than that of Nova1_L_ variants on the migration of CRC cells (Fig. 4a, bar chart). In addition, results of the RT-PCR, RT-qPCR, and immunoblot analyses showed that overexpression of RBM4 enhanced expression of E-cadherin and led to a concomitant decrease in N-cadherin (Fig. 4b, lanes 2 and 6; bar chart) which are critical factors involved in the epithelial-to-mesenchymal transition (EMT)^23^. Overexpression of Nova1 isoforms exerted opposite effects on diminishing E-cadherin expression and the concomitant upregulation of N-cadherin levels in CRC cells (Fig. 4b, lanes 3, 4, 7, and 8). These results suggested that RBM4 and Nova1 proteins potentially constitute an antagonistic mechanism that manipulates the EMT-related pathway in CRC cells.

### RBM4 and Nova1 exhibit opposite effects on splicing profiles of SRSF6

**Figure 5:**
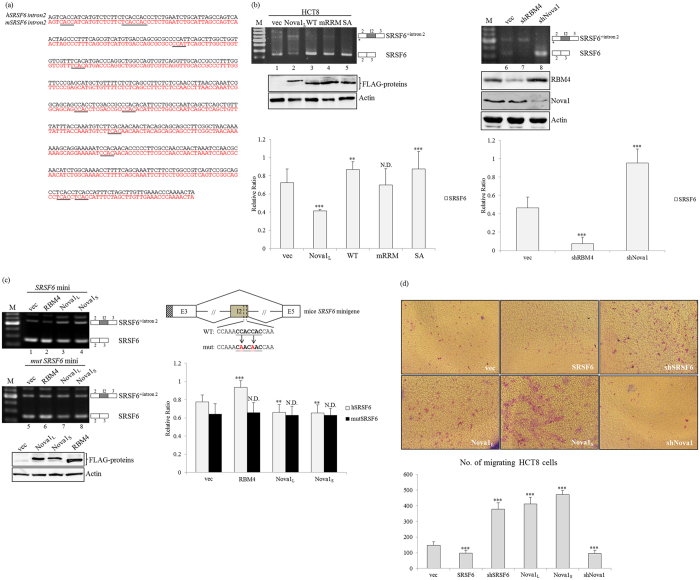
RBM4 and Nova1 variants exhibit opposite effects on splicing profiles of the *SRSF6* gene. (**a**) The diagram presents the complete identity between sequences of the 602-bp human and mouse *SRSF6* intron 2 fragments. The responsive elements of Nova1 are underlined. (**b**) HCT-8 cells were transfected with overexpressing or targeting vectors, followed by total RNA and lysate extraction. Splicing profiles of *SRSF6* transcripts were analyzed with specific primer sets. Immunoblot assays of indicated proteins were analyzed with specific antibodies. The bar graph shows relative levels of *SRSF6* coding transcripts in three independent experiments. (**c**) The *SRSF6* minigene and derived mutant were cotransfected with expressing vectors into HCT-8 cells. Spliced transcripts of the *SRSF6* minigenes were analyzed as described in Fig. 3b. The bar graph shows relative levels of *SRSF6* coding transcripts in three independent experiments. The signal densities were analyzed using TotalLab Quant Software, and the quantitative results are shown as the mean ± STD (*p < 0.05; **p < 0.01; ***p < 0.005). The gels shown in this figure were run under the same conditions and were not artificially manipulated. (**d**) The motility of HCT-8 cells transfected with SRSF6 or Nova1 variant-expressing vectors or targeting vectors was analyzed using an *in vitro* migration assay and the counting results of three independent experiments are presented in a bar chart as the mean ± STD.

In addition to *Nova1*, differential splicing profiles of the *SRSF6* gene in CRC tissues compared to adjacent normal tissues were revealed using RNA-seq (Fig. 1b, lower panel) and RT-PCR analyses (Fig. 2a). Nova1 was documented to facilitate the inclusion of mouse *SRSF6* intron 2, which shares complete identity with the human *SRSF6* gene (Fig. 5a), in a YCAY element-dependent manner^16^. Splicing profiles of human *SRSF6* were therefore examined with the overexpression or depletion of Nova1 or RBM4 in CRC cells. As shown in Fig. 5b, the presence of RBM4-WT and the derived S309A mutant induced a relatively high level of authentic *SRSF6* transcripts (Fig. 5b, lanes 3 and 5), whereas overexpression of the Nova1_L_ variant reduced the relative expression of coding *SRSF6* transcripts in HCT8 cells (Fig. 5b, lane 2). The splicing pattern of *SRSF6* was sustained with overexpression of the RBM4 RNA recognition motif-mutant containing four mutations (Y37A, F39A, Y113A, and F115A) (Fig. 5b, lane 4). Inversely, ablation of endogenous RBM4 mediated upregulation of *SRSF6*^*+intron 2*^ transcripts (Fig. 5b, lane 7), whereas upregulated generation of *SRSF6* transcripts was observed in Nova1-knockdown HCT8 cells (Fig. 5b, lane 8). To validate the inference of the Nova1-regulated mechanism on *SRSF6* splicing, *in vivo* splicing assays were conducted with the *SRSF6* minigene and the previously established derived mutant^16^. Increases in intron 2-skipped transcripts generated from the SRSF6 minigene were observed with RBM4a overexpression compared to empty-vector transfectants (Fig. 5c, lanes 1 and 2). In contrast, relatively high expression levels of *SRSF6*^*+intron 2*^ transcripts were noted in Nova1 variants-overexpressing HCT8 cells (Fig. 5c, lanes 3 and 4). The mutant minigene harboring the CA substitution within the CCAC motif (Fig. 5c, diagram) showed an insubstantial response to overexpression of the RBM4 or Nova1 isoforms (Fig. 5c, lanes 5 ~ 8).

SRSF6 was reported to program cancer-related splicing profiles, including *CD44* and *corticotropin-releasing hormone receptor* genes, which subsequently inhibit the progression of cancer cells^24^,^25^. Results of the *in vitro* migration assays and statistical analyses showed that SRSF6 overexpression (Fig. 5d, SRSF6) diminished the migratory activity of HCT8 cells, whereas SRSF6-knockdown (Fig. 5d, shSRSF6) cells exhibited more-substantial migration compared to empty-vector transfectants. In contrast, ablation of Nova1 abrogated the progression of CRC cell lines (Fig. 5d, shNova1), which was consistent with previous reports^13^. These results suggested the hierarchical role of the RBM4a-Nova1 interplay in manipulating splicing profiles of the *SRSF6* gene and its physiological significance in CRC cells.

### The RBM4-regulated splicing cascade programs splicing profiles and effects of the VEGF gene

**Figure 6:**
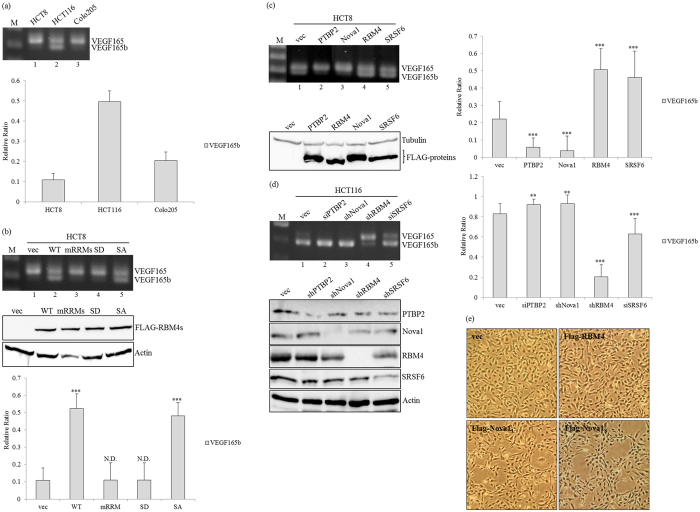
The RBM4-initiated splicing cascade modulates splicing profiles of *vascular endothelial growth factor (VEGF*) transcripts, which is relevant to the angiogenic activity of colorectal cancer (CRC) cells. (**a**) Total RNAs prepared from CRC-derived cell lines were analyzed by an RT-PCR with a specific primer set against *VEGF165* transcripts. The bar graph shows relative levels of *VEGF165b* in three independent experiments. (**b**) HCT-8 cells were transfected with overexpressing-RBM4 and derived mutants, followed by total RNA and lysate extraction. Splicing profiles of *VEGF165* transcripts were analyzed with specific primer sets. Immunoblot assays of indicated proteins were analyzed with specific antibodies. The bar graph shows relative levels of *VEGF165b* in three independent experiments. (**c**) Splicing profiles of *VEGF165* transcripts in HCT-8 cells transfected with expressing vectors of splicing regulators were analyzed as described in the last panel. The bar graph shows relative levels of *VEGF165b* in three independent experiments. The overexpressing proteins and loading control were monitored with specific antibodies. (**d**) HCT-116 cells were transfected with targeting vectors of various splicing regulators, followed by total RNA and lysate extraction. Splicing profiles of *VEGF* transcripts and indicated proteins were examined with specific primer set and antibodies. The bar graph shows relative levels of *VEGF165b* in three independent experiments. The signal densities were analyzed using TotalLab Quant Software, and quantitative results are shown as the mean ± STD (*p < 0.05; **p < 0.01; ***p < 0.005). The gels shown in this figure were run under the same conditions and were not artificially manipulated. (**e**) Angiogenic activities of HCT-8 cells transfected with RBM4 or Nova1 variant-expressing vectors were analyzed using an *in vitro* tubular formation assay. Cell images were photographed with a microscope system, and the angiogenesis of each transfectant was quantified from four random fields of each well.

In addition to progressive signatures, active angiogenesis is another hallmark of various cancers, including CRC cells^26^. Both the expression and splicing profiles of VEGF isoforms regulate the angiogenic capability of colorectal carcinomas^27^. Proangiogenic VEGF165 and antiangiogenic VEGF165b isoforms are generated from the same gene through alternative splicing mechanisms^28^. Previous studies documented that the presence of SRSF6 favors the selection of VEGF exon 8b by directly interacting with the distal 3′ splice site, which encodes VEGFb variants^29^. Even though predominant expression of *VEGF165* was observed in CRC tissues^30^, differential splicing profiles of *VEGF* were validated in distinct CRC cell lines. Except for adenocarcinoma-derived cell lines, *VEGF165b* transcripts were only generated in HCT116 carcinoma cells (Fig. 6a, lane 2), which may be related to expression profiles of splicing regulators. The overexpression of RBM4-WT and RBM4-SA proteins (Fig. 6b, lanes 2 and 5), but not the RRM-mutant (Fig. 6b, lane 3), induced the relative levels of *VEGF165b* transcripts. The cytoplasmic-enriched RBM4 mutant harboring serine-to-aspartate (S309D) substitution exerted an insubstantial effect compared to the RRM mutant on the splicing profile of the *VEGF* gene (Fig. 6b, lane 4). Moreover, overexpressing the PTBP2 and Nova1 proteins (Fig. 6c, lanes 2 and 3) exerted opposite effects as that of RBM4 and SRSF6 (Fig. 6c, lanes 4 and 5) of further enhancing relative levels of *VEGF165* transcripts. Inversely, the majority of *VEGF165* transcripts were generated with depletion of the endogenous PTBP2 and Nova1 proteins (Fig. 6d, lanes 2 and 3), whereas relative levels of *VEGF165b* transcripts were induced in RBM4- and SRSF6-knockdown HCT116 cells (Fig. 6d, lanes 4 and 5). Collectively, splicing profiles of the *VEGF* gene were meticulously manipulated by the relative expressions of RBM4, Nova1, and SRSF6. The physiological significance of the RBM4 and Nova1 proteins on angiogenic activity was evaluated by conducting *in vitro* tubule formation assays with HUVECs. As expected, overexpression of Nova1 variants induced the fusion of neighboring HUVECs to form several capillary-like lumens by 24 h post-transfection (Fig. 6e, Flag-Nova1), whereas the cellular morphology remained unchanged in RBM4-transfected cells compared to empty-vector transfectants. Taking these results together, the RBM4-regulated splicing cascade constitutes a novel mechanism involved in the angiogenic activity of CRC cells.

## Discussion

Imbalanced splicing events are reportedly relevant to carcinogenic signatures, including immortality, progression, and angiogenesis^31^,^32^,^33^. In this study, we performed deep RNA-seq to reveal the CRC-related splicing cascade composed of the *Nova1, SRSF6*, and *VEGF* genes. The molecular mechanisms involved in programming the splicing profiles of these genes were further investigated. We next observed a marked influence of this splicing cascade on the progression and angiogenesis of CRC-derived cell lines.

We previously reported the impact of the RBM4-regulated splicing cascade on the migration, invasion, and metabolic signatures of CRC cells^13^. Reduced expression of RBM4 was documented to be related to a poor prognosis of gastric cancer patients^34^. To determine the possible influence of RBM4 on cellular processes, a transcriptome-wide approach was conducted to identify the putative target of RBM4^35^. To the best of our knowledge, this is the first report of the differential splicing profiles of *Nova1* transcripts revealed in CRC tissues compared to adjacent normal counterparts. The Nova1 protein was first identified as a splicing factor predominantly expressed in neurons of the central nervous system^36^. Development of high-throughput sequencing coupled with RNA crosslinking and immunoprecipitation demonstrated the previous prediction that Nova1 regulated alternative splicing events in a YCAY-dependent manner^36^,^37^. More than 800 neuron-specific splicing candidates of Nova1 were identified with this methodology, which implies the potential function of Nova1 in neurogenesis^38^. In our previous study, speckle-enriched localization of the Nova1_S_ variant was highly relevant to its potent influence on modulating brown adipocyte-associated splicing networks, which further enhanced brown adipogenesis^16^. Recent studies demonstrated that an increase in Nova1 expression functioned as a pro-oncogene involved in the proliferation, progression, and immortality of various cancer cells^21^,^22^,^39^. The overexpression of Nova1 variants was noted to mediate the cadherin switching from E-cadherin to N-cadherin expression, which enhanced the migration of HCT8 cells. The differential splicing profile of *Nova1* might constitute a molecular mechanism in modulating the cadherin switching in other CRC cells, such as HCT116, although more convincing result is required to demonstrate the speculation. The prominent effect of the Nova1_S_ isoform on enhancing the migration of CRC cells could be also achieved by modulating other alternative splicing event. The differential expression network of splicing regulator in distinct cancer cells might modulate the impact of Nova1 variants on carcinogenic signature by reprogramming the splicing profiles of *Nova1*. Nevertheless, the characterization of Nova1-specific candidate is critical for determining its impact in the migration or other carcinogenic signature of CRC.

Autoregulation constitutes a common mechanism of controlling splicing profiles of splicing factors including Nova^19^,^36^. Nova1 variants were reported to exhibit differential effects on the utilization of its own exon 4 in neurons, pancreatic β-cells, and brown adipocytes^16^,^19^,^36^. Nevertheless, RBM4 was demonstrated to play a hierarchical role in controlling splicing profiles of the *Nova1* gene during brown adipogenesis^16^. However, differential influences of Nova1 variants on regulating the human *Nova1* minigene and the derived mutant were insubstantial compared to those of the mouse *Nova1* reporter. The shortened CU element interrupted by one guanine residue may lessen the influence of RBM4, which subsequently strengthened the impact of Nova1 variants. Nevertheless, the interplay between RBM4 and Nova1 constituted a molecular mechanism involved in *Nova1* splicing, which was widely identified in different tissues and species.

The alternative splicing-coupled NMD pathway is reportedly a common mechanism for modulating expressions of SR proteins and hnRNP family members^40^,^41^. Results of mRNA-seq identified the dominant presence of non-coding *SRSF6* transcripts containing an in-frame premature stop codon within the 268-bp intronic fragment in total RNAs extracted from CRC tissues. The complete identity between the included intron 2 within human and mouse *SRSF6* genes implies that the conserved mechanism contributes to the generation of this non-coding *SRSF6* transcript, which mediated the reduced protein level of SRSF6 in CRC tissues compared to adjacent normal tissues. Although expression levels of SRSF6 were documented to be correlated with the progressive activity of various cancers, relative expression levels of SRSF6 in normal and malignant tissues and its physiological significance were previously debated^7^,^42^. An elevated *SRSF6* gene copy number was previously validated using public databases^7^. In our present study, relative expressions of both *SRSF6* transcripts and coding protein were examined with paired tissue samples of anonymous CRC patients. Moreover, the differential splicing profiles of the *SRSF6* gene in distinct CRC cell lines derived from adenocarcinomas or carcinomas also suggested its potential application as a classification marker of CRC patients. Consistently, the upregulated expression of SRSF6 diminished the progression or angiogenesis of cancer cells^24^,^42^.

**Figure 7:**
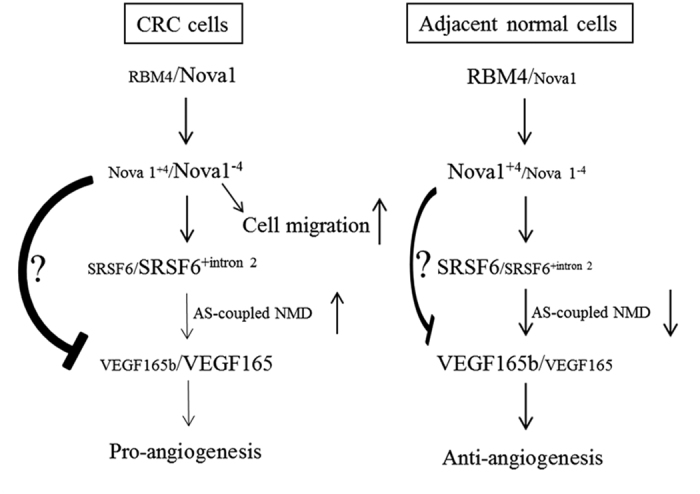
The RBM4-regulated splicing cascade was correlated with the progression of colorectal cancer (CRC) cells. In CRC cells, the downregulated level of RBM4 leads to a concomitant increase in the expression of total *Nova1* transcripts and relative levels of *Nova1*^−*4*^, which subsequently and coordinately strengthened the influence of Nova1 on its splicing target, including SRSF6. Upregulated Nova1 variants enhanced the angiogenic activity of CRC cells by indirectly inducing relative expressions of *VEGF165* transcripts, which more greatly enhanced the angiogenesis of CRC cells.

Our studies show that RBM4 regulates the expression level and splicing profiles of *Nova1* through multilayer posttranscriptional controls, including mRNA stability and alternative splicing^13^. In CRC tissues and cells, reduced RBM4 expression led to relatively high levels of *Nova1*^−*4*^ transcripts which were greater than upregulated expressions of total *Nova1* mRNAs, subsequently enhancing the migratory activity of CRC cells. *Nova1*^−*4*^ variants demonstrably exerted a prominent effect on mediating the AS-coupled NMD of the *SRSF6* gene, which in turn led to a reduction in antiangiogenic *VEGF165b* variants (Fig. 7). In conclusion, our study first identified CRC-associated splicing profiles of the *Nova1* and *SRSF6* transcripts in paired CRC tissues using mRNA-seq and *in vivo* splicing assays. The molecular mechanisms involved in this RBM4-initiated splicing cascade were further investigated with CRC-derived cells. Correlations between migratory or angiogenetic activities and this splicing cascade, composed of RBM4, Nova1, SRSF6, and VEGF, were also demonstrated. To bring the comprehensive scope into the influence of SRSF6 or Nova1 on CRC, the potential candidate of these splicing factor is worthy of further identification. To date, the relatively high level of *Nova1*^−*4*^ or reduced *SRSF6* expression could be considered as the classificatory or prognostic markers of CRC. Moreover, reprogramming of the CRC-associated splicing cascade can be applied to therapeutic strategies targeting carcinogenic signatures.

## Methods

### Ethics statement of patient samples and cell culture

The human colorectal tumor samples (*n* = 20) with informed consent were requested as anonymous specimens from the Joint Biobank at Taipei Medical University. All experiments with clinical specimens were performed in accordance with relevant guidelines and regulations. This study was approved by the Joint Institutional Review Board of Taipei Medical University (approval no. 201409044). All of the recommendations of the *Declaration of Helsinki* for biomedical research involving human subjects were followed. HCT-8, Colo205, and HCT-116 cells were cultured in RPMI-1640 medium supplemented with 10% fetal bovine serum (FBS), 600 mg/ml glutamine, 100 U/ml penicillin, and 100 mg/ml streptomycin (Invitrogen, Camarillo, CA, USA). Human umbilical vein epithelial cells (HUVECs) were a kind gift from Dr. Wen-Sen Lee (Taipei Medical University, Taipei, Taiwan). HUVECs were cultured in medium (M)-199 supplemented with 10% FBS, 10 U/ml heparin sodium, 15 mg/L of endothelial cell growth factor, and antibiotics.

### RNA extraction and complementary (c)DNA library construction and sequencing

Total RNAs were prepared from clinical tissues using the PureLink RNA mini kit (Invitrogen) according to the manufacturer’s protocol. To minimize the contribution of amplified reads that are aligned with intergenic DNA fragments, extracted RNAs were treated with DNase I (Promega, Madison, WI, USA) prior to library construction. The integrity and quantity of the RNAs for deep RNA-seq were assessed using an Agilent 2100 Bioanalyzer (Agilent Technologies, Redwood, CA, USA). Poly(A) messenger (m)RNAs were enriched from 8 μg total RNAs with a high integrity number (RIN > 8.0) using oligo (dT)-based affinity matrices, followed by library construction using the NEB Next Ultra RNA Library Prep Kit from Illumina (NEB, Ipswich, MA, USA) according to the manufacturer’s instructions. The prepared libraries were sequenced on an Illumina NextSeq 500 platform, and ~150-bp paired-end reads were generated.

### Read mapping and transcript annotation and quantification

Preliminary reads were cleaned by trimming the adapter sequences and removing poly-N or low-quality sequences (Q < 20). Filtered reads were aligned to the mouse reference genome (GRCm37) using the Tophat v2.0.9 program (available where ? ). Tolerance parameters were the default setting to allow mismatches of fewer than two bases. Transcriptome assemblies of aligned reads were generated using the Cufflink program. Mutant loci within the assembled transcripts were identified using SAM tools. These transcriptome assemblies generated from individual samples were merged together using the Cuffmerge utility to provide a standard for estimating transcript levels in each condition. Expression levels and the statistical significance of the merged assemblies were calculated using the Cuffdiff analysis.

### Plasmid construction

The pUNova1 and pUSRSF6 minigene reporters were constructed as previously described^16^. The human Nova1 and a derived mutant of Nova1 or SRSF6 minigenes harboring substituted nucleotides within a cassette exon or intron were constructed using the QuikChange site-directed mutagenesis system (Stratagene, Amsterdam, the Netherlands). Expression vectors for the human SRSF6 gene were constructed by placing the coding sequence in-frame into the p3XFLAG-CMV14 vector (Sigma, St. Louis, MO, USA). The human SRSF6 coding region was polymerase chain reaction (PCR)-amplified with the reverse-transcription (RT) product prepared from the total RNA of HCT8 cells as the template and then inserted into *Hind* III/*Not* I sites of the vector.

### Transient transfection, and RT-PCR and quantitative RT-(q)PCR assays

CRC-derived cell lines were grown to 60 ~ 70% confluence, and the plasmid was transfected using Lipofectamine 3000 according to the manufacturer’s protocol (Invitrogen). Total RNAs were extracted using a PureLink RNA mini kit at 24 h post-transfection (Invitrogen). RNAs prepared from clinical tissues or cultured cells (1 μg) were reverse-transcribed using SuperScriptase III (Invitrogen) in a 10-μl reaction and subjected to a PCR analysis with gene-specific primer sets (Supplementary Table 1). An RT-qPCR was performed with SYBR green fluorescent dye and gene-specific primer sets (Supplementary Table 2) using an ABI One Step™ PCR machine (Applied Biosystems, Foster City, CA, USA). The relative mRNA level was quantitated by the ΔΔ-Ct method, and normalized to the level of GAPDH mRNA.

### Immunoblot assay

The immunoblot analysis was performed using an enhanced chemiluminescence (ECL) system (Millipore, Billerica, MA, USA), and results were monitored using the LAS-4000 imaging system (Fujifilm, Tokyo, Japan). Primary antibodies used in this study included polyclonal anti-RBM4^16^, monoclonal anti-PTBP2 (Abnova, Taipei, Taiwan), monoclonal anti-SRSF6 (EMD, Millipore), monoclonal anti-Nova1 (Abnova), monoclonal anti-actin (Millipore), monoclonal anti-α-tubulin (Abcam, Cambridge, UK), monoclonal anti-E-cadherin (Abcam), monoclonal anti-N-cadherin (Abcam), and monoclonal anti-FLAG M2 (Sigma-Aldrich, St. Louis, MO, USA). Signal intensities were evaluated using TotalLab Quant Software (where available?).

### *In vitro* migration and tubule formation assays

HCT-8 cells (5 × 10^4^ cells/well) transfected with distinct vectors or targeting vectors were seeded in the top chamber containing a polycarbonate membrane (8-μm pore, Corning, Cambridge, MA, USA). The lower chamber contained complete culture medium which served as a chemoattractant. After 24 h, the inner membrane was scraped with a swab, and cells which had migrated to the lower side of the membrane were fixed with 4% paraformaldehyde and stained with a Giemsa solution for counting. For the *in vitro* tubular formation assay, 5 × 10^4^ HUVECs transfected with expressing or targeting vectors were suspended in 500 μl of complete M-199 medium and then seeded on each well of 6-well plates coated with Matrigel. After incubation at 37 °C for 24 h, each well was photographed with an Olympus IX81 microscope (Olympus, Tokyo, Japan). Tubule formation was quantified from four random fields per well.

### Statistical analyses

Student’s *t*-tests were performed to determine the significance of the above-described experiments. *p* < 0.05 was considered statistically significant.

## Additional Information

**How to cite this article:** Lin, J.-C. *et al*. The impact of the RBM4-initiated splicing cascade on modulating the carcinogenic signature of colorectal cancer cells. *Sci. Rep.* **7**, 44204; doi: 10.1038/srep44204 (2017).

**Publisher's note:** Springer Nature remains neutral with regard to jurisdictional claims in published maps and institutional affiliations.

## Supplementary Material

Supplemental Table
