# Supplementary material for: The impact of the RBM4-initiated splicing cascade on modulating the carcinogenic signature of colorectal cancer cells
Source: Sci Rep. 2017 Mar 9;7:44204. doi: 10.1038/srep44204 (PMC5343574; doi:10.1038/srep44204)
Supplement: Supplemental Table [file srep44204-s1.pdf]

The impact of the RBM4-initiated splicing cascade on modulating the carcinogenic signature of colorectal cancer cells

Jung-Chun Lin[1], Yuan-Chii Gladys Lee[2,*], Yu-Chih Liang[1,*], Yang C. Fann[3], Kory R. Johnson[3], Ying-Ju Lin[4]

[1]School of Medical Laboratory Science and Biotechnology, College of Medical Science and Technology, Taipei Medical University, Taipei, Taiwan

[2]Graduate Institute of Biomedical Informatics, Taipei Medical University, Taipei, Taiwan

[3]Information Technology and Bioinformatics Program, Division of Intramural Research, National Institute of Neurological Disorders and Stroke, National Institutes of Health, Bethesda, MD, USA

[4]School of Chinese Medicine, China Medical University, Taichung, Taiwan

[*]These authors contributed equally to this work.

Running Title: RBM4a modulates CRC-related splicing events

To whom correspondence should be addressed: Jung-Chun Lin

School of Medical Laboratory Science and Biotechnology, College of Medical Science and Technology, Taipei Medical University

250 Wu-Hsing Street, Taipei 11031, Taiwan

Telephone: +886-2-27361661 ext. 3330

Fax: +886-2-27324510

E-mail: lin2511@tmu.edu.tw

Supplemental Table 1. PCR Primers.

Gene	Forward	Reverse
Nova1-AS	tgccccaaaatgtggccaagaca	agccttcacagtagcacctccct
Nova1-Total	tctgatcccatgaccacctcca	ctgctggaaaggccgcaaca
SRSF6-AS	acgacgccgtttacga	ttaaatcttgccaactgcac
SRSF6 mini (F2)	atccagctgagctttatgctgtatttg	atcctgcagtaagggcaagtcaaatagac
Gapdh	cggagtcaacggatttggtcgtatg	agccttctccatggtggtgaagac
VEGF165-AS	ttgtacaagatccgcagacg	gttctgtatcagtctttcctgg
SV40	ttttggaggcctaggctttt	
E-cadherin	tggaggaattcttgctttgc	cgtacatgtcagccagcttc
N-cadherin	ggcttaatggtgattttgctc	ctcaagtcatagtcctggtc

Supplemental Table 2. Quantitative RT-PCR Primers.

Gene	Forward	Reverse
Nova 1	tgagggaacaggtggagaac	agctggacacggagcttttta
E-cadherin	cagaaagttttccaccaaa	aaatgtgagcaattctgctt
N-cadherin	cgagccgcctgcgctgccac	cgctgctctccgctccccgc
Gapdh	aaggtcatcccagagctgaa	ctgcttcaccaccttcttga
